# Influence of Congested Match Schedules, Pre-Match Well-Being and Level of Opponents on Match Loads during World Rugby Women’s Sevens Series

**DOI:** 10.3390/ijerph182212132

**Published:** 2021-11-19

**Authors:** Daniele Conte, Aristide Guerriero, Corrado Lupo, Ademir Felipe Schultz Arruda, Paulius Kamarauskas

**Affiliations:** 1 Latvian Academy of Sport Education, LV-1006 Riga, Latvia; 2 Brazilian Rugby Confederation, São Paulo 04575, SP, Brazil; aristide.guerriero@gmail.com (A.G.); felipe.schultz@brasilrugby.com.br (A.F.S.A.); 3 NeuroMuscular Function Research Group, Department of Medical Sciences, School of Exercise & Sport Sciences (SUISM), University of Turin, I-10125 Turin, Italy; corrado.lupo@unito.it; 4 Institute of Sport Science and Innovations, Lithuanian Sports University, LT-44221 Kaunas, Lithuania; paulius.kamarauskas@lsu.lt

**Keywords:** GPS, external load, RPE, internal load, wellness, congested fixture

## Abstract

This study aimed at assessing (1) the effect of congested match schedules on match loads and well-being as well as (2) pre-match well-being and level of opponents on match loads in elite women’s rugby sevens. Eleven players of the Brazilian women’s rugby sevens national team were investigated across three 2019-20 HSVC World Rugby Women’s Seven Series tournaments to assess: (1) within-tournament match-to-match changes in various external and internal match load measures; (2) daily changes in players’ well-being collected before the commencement of a tournament (day one) and during or post-tournament (day two to day four); and (3) the effect of pre-match well-being and level of opponents (high vs. low level) on match loads. Results revealed no between-match significant differences (*p* > 0.05) in most of the investigated match load measures. A congested match schedule negatively affected perceived fatigue (*p* < 0.001), muscle soreness (*p* = 0.004) and overall wellness (*p* < 0.001), with post hoc analyses showing decreased values on day four compared to previous days (small-to-moderate effect sizes). Finally, pre-match well-being and level of opponents did not affect match loads (*p* > 0.05). These results highlighted the necessity to embrace a multidimensional approach when adopting monitoring systems in elite women’s rugby sevens during tournaments and to consider various contextual factors possibly affecting match loads, besides those investigated.

## 1. Introduction

Women’s rugby sevens is a team sport characterized by high physical and physiological demands [1,2]. Elite national women’s rugby sevens teams compete within the HSBC World Rugby Women’s Seven Series, which includes numerous international tournaments encompassing several matches in close succession (S1) (https://www.world.rugby/sevens-series/calendar. Access date on 25 July 2021). Understanding the match loads imposed during these international tournaments seems essential to prescribe an adequate training load and implement sound recovery strategies [3,4,5]. In fact, a congested match schedule might induce a decrement in performance across consecutive matches.

A previous study assessing the influence of a congested match schedule during the World Rugby Women’s Seven Series on a team playing five matches across a two-day tournament showed no statistical differences in match load quantified using GPS devices (total distance covered as well as distance covered at low, medium and high intensity) [5]. Similarly, another investigation showed that a two-day tournament encompassing four–six matches induced no substantial performance changes in national-level players [3]. However, a congested match schedule has been found to induce an impairment in perceived well-being, fatigue, mood, general muscle soreness and stress levels [5], as well as a two-fold increase in creatine kinase (CK), which is one of the main markers of muscle damage [3]. It should be noted that non-top teams are usually involved in a lower number of matches compared to the teams investigated in previous studies, competing in no more than two matches per day. This lower match frequency during tournaments might provide different results compared to previous investigations in terms of physical performance and well-being changes. To date, no previous investigation has assessed the effect of a tournament schedule inducing lower match frequencies in women’s rugby sevens. Therefore, the assessment of changes in match loads and well-being is warranted.

A congested match schedule is not the only factor potentially influencing match loads in rugby sevens. Intuitively, increased fatigue levels and decreased overall wellness due to matches played in close succession might reduce the match loads experienced during matches in tournaments. However, while an influence of match loads on well-being and CK in elite women’s rugby sevens players has been shown [3,5], no previous studies have assessed whether pre-match well-being also influences match loads. This research question seems fundamental for practitioners since, knowing the pre-match players’ well-being, it can be possible to organize the best line-up for upcoming matches. Thus, the assessment of pre-match well-being on match loads is necessary to provide useful practical applications.

It should be noted that other factors may play a substantial role in affecting match loads in women’s rugby sevens during tournaments. Indeed, a previous study on men’s rugby sevens showed that players are likely to perform greater peak periods of running against higher-ranked opponents, possibly indicating a potential relationship between the level of opponents and match loads [6]. However, to the best of our knowledge, no previous study has assessed the effect of the level of opponents on women’s rugby sevens during tournaments. This contextual factor, monitored in combination with match schedules (congested or not) and pre-match well-being, might provide new insights about variables affecting match loads in women’s rugby sevens. Therefore, the aims of this study were to assess the effect of: (i) congested match schedules on match loads and well-being as well as (ii) pre-match well-being and level of opponents on match loads in elite women’s rugby sevens.

## 2. Materials and Methods

### 2.1. Design

A repeated-measures observational study was designed to assess the effect of a congested match schedule, pre-match well-being and level of opponents on match loads during three rounds of the HSVC World Rugby Women’s Seven Series 2019–2020 season (round 2: Dubai, United Arab Emirates, 5–7 December 2019; round 3: Cape Town, South Africa, 13–15 December 2019; and round 4: Hamilton, New Zealand 25–26 January 2020). Each investigated round consisted of three pool matches and one playoff match for a total of 12 investigated matches (11 losses by 22.3 ± 13.1 points; 1 win by 2 points).

**Figure 1:**
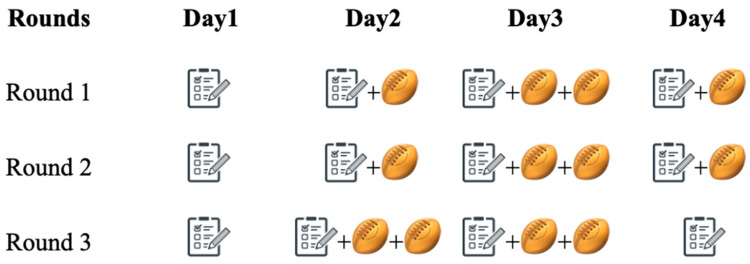
Study design. 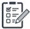 indicates the well-being questionnaire. 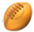 indicates the investigated matches across the three rounds.

The data collected during the three rounds are displayed in Figure 1. The well-being values were collected daily at the same time of the day (~8:15 a.m.), in the day before the commencement of the tournament (day 1) and in the following match days, before playing the first match of the day (Figure 1). In particular, one or two daily matches were played between day 2 and day 4 (Figure 1). Specifically, in round 2 and 3, players were involved in one match in day 2 and day 4, while two matches were played on day 3. In round 4, players were involved in two daily matches on day 2 and day 3. In the case of two matches being played on the same day, only the match loads of the first match were considered in the analysis when considering the effect of pre-match well-being, to avoid any possible fatigue effect deriving from the first daily match.

Across the 3 tournaments, the investigated team played against eight of the competing teams in the 2020 championship. However, it should be considered that the variable level of opponents was considered in combination with pre-match well-being in our analysis. Therefore, in the case of two matches being played on the same day, only the match load of the first match was considered for the analysis in addition to its respective pre-match well-being and opponent level. Therefore, a total of 7 opponent teams were included in this study.

### 2.2. Participants

Sixteen players of the Brazilian women’s rugby sevens national team participating in 2019-20 HSVC World Rugby Women’s Seven Series season were recruited for this study. Five players were excluded from the analysis due to not participating in at least a full tournament and playing four matches. As a consequence, a study sample of 11 players (mean ± standard deviation, age: 24.3 ± 3.3 year; stature: 166.1 ± 7.2 cm; body mass: 66.1 ± 7.4 kg; and training experience: 6.3 ± 1.8 year) met the inclusion criteria and was involved in the final analysis. Since this study encompasses a repeated-measures design (i.e., players are involved in more than one match), a final sample of 72 individual match samples across the investigated tournaments was considered. All investigated players were adults (>18 year.) and familiarized with the aims, procedures, requirements and benefits of the study prior to the beginning of data collection, before they were asked to provide a written consent of participation. Ethical approval was obtained from the Ethics Committee of the Latvian Academy of Sport Education (ref. number: 333/42813).

### 2.3. Procedures

#### 2.3.1. External Load Measures

GPS units (Catapult OptimEye X4, Catapult Innovations Melbourne, Australia) sampling at 10 Hz with integrated accelerometers and gyroscopes sampling at 100 Hz to calculate instantaneous dynamic movement demands were used to measure external load measures. The reliability and validity of this device have been previously identified for field-based sports [7]. Approximatively 30 min before the investigated matches, GPS units were activated and positioned in the jersey pocket between shoulder blades for each player. After the completion of each match, collected data were downloaded from each GPS unit and then processed via OpenField software (version 1.18, Catapult Innovations, Melbourne, VIC, Australia). External load data were used to quantify match intensity by measuring their relative values per minute: total distance (TD/min), standing or walking at 0–6.0 km·h^−1^ (walking/min), jogging at 6.1–12.0 km·h^−1^ (jogging/min), cruising at 12.1–14.0 km·h^−1^ (cruising/min), striding at 14.1–18.0 km·h^−1^ (striding/min), high-intensity running at 18.1–20.0 km·h^−1^ (HIR/min), sprinting at >20.1 km·h^−1^ (sprint/min), number of accelerations at >1.8 m·s^−2^ (ACC/min) and number of decelerations at <−1.8 m·s^−2^ (DECEL/min) [8,9].

#### 2.3.2. Internal Load Measures

Individual session rating of perceived exertion (sRPE) was used as the indicator of match load intensity. sRPE values were collected within 30 min after the end of the investigated matches using a 10-point RPE scale (CR-10) [10]. sRPE match load (sRPE-ML), which was used as a measure of internal load volume, was then calculated by multiplying sRPE values by the match duration in minutes. Match durations were considered as time spent on the field excluding between-halves break and bench time [5].

#### 2.3.3. Well-Being Questionnaire

A five-point Likert well-being questionnaire (scores 1–5), which was previously used in team sport athletes [11,12,13], was collected daily using cloud-based software (Google Docs) to assess a player’s perceived fatigue, sleep quality, general muscle soreness, stress levels and mood. The individual, overall wellness of each player was then calculated by summing the scores across each item assessed [11,13].

#### 2.3.4. Level of Opponents

Since this study was run retrospectively, the level of opponents was assessed based on the points gained by each opponent team for the final standing at the end of the 2020 championship. Successively, a k-means cluster analysis was performed to classify the level of opponents in two clusters (high vs. low), resulting in four teams being in the high-level and three teams being in the low-level cluster.

### 2.4. Statistical Analyses

Data are presented as mean and standard deviation, and all well-being data were log-transformed before the analysis. For the first aim of this study, separate linear mixed models (LMMs) were used for each match load measure, using a match as a fixed effect and a player as a random effect. Moreover, LMMs were used with well-being items and overall wellness as dependent variables, the day as a fixed effect and a player as a random effect. In the case of statistically significant differences, post hoc analyses were used for pairwise comparisons with Bonferroni corrections. For statistically significant pairwise comparisons, effect sizes (ESs) with 95% confidence intervals were calculated and interpreted as trivial < 0.20, small = 0.20–0.59, moderate = 0.60–1.19, large = 1.20–1.99 and very large ≥ 2.00 [14].

Successively, LMMs were used with load measures as dependent variables, the level of opponents and the five well-being items as fixed effects and a player as a random effect. The same analysis was also run using the overall wellness score instead of the five well-being items. All random effects were considered with a random intercept and a fixed slope. An alpha level of *p* < 0.05 was set a priori for statistical significance. All data were analyzed using Jamovi software (version 1.2.27, 2020).

## 3. Results

The analysis of match-to-match variation across the investigated rounds showed significant changes in striding/min only (*p* = 0.021), while the other external load measures showed no significant differences (Table 1). Post hoc analysis revealed significantly lower striding/min values during match three when compared with match one (*p* = 0.037, ES = 0.71 (95% CI = 0.13; 1.27), moderate).

A congested match schedule significantly affected fatigue (*p* < 0.001), sleep quality (*p* = 0.043), muscle soreness (*p* = 0.004) and overall wellness (*p* < 0.001) (Table 2). Post hoc analyses revealed that lower fatigue values in day four compared to day one (*p* = 0.004, ES = 0.76 (95% CI = 0.20; 1.32), moderate), day two (*p* < 0.001, ES = 0.99 (95% CI = 0.39; 1.57), moderate) and day three (*p* = 0.004, ES = 0.76 (95% CI = 0.20; 1.32), moderate). No differences in post hoc analyses for pairwise comparisons were shown for sleep quality (*p* > 0.05). Considering muscle soreness, post hoc analyses revealed lower values in day four compared to day one (*p* = 0.005, ES = 0.79 (95% CI = 0.28; 1.36), moderate) and day two (*p* = 0.030, ES = 0.66 (95% CI = 0.11; 1.21), moderate). Finally, overall wellness post hoc analyses revealed lower values in day four compared to day one (*p* < 0.001; ES = 0.95 (95% CI = 0.36; 1.52), moderate), day two (*p* < 0.001, ES = 1.04 (95% CI = 0.44; 1.63), moderate) and day three (*p* = 0.017, ES = 0.59 (95% CI = 0.04; 1.14), small).

The results of the effect of well-being and level of opponents on match loads are shown in Table 3 and Table 4. Results revealed that match loads are not influenced (*p* > 0.05) by pre-match well-being items when considered separately (Table 3) or summed as overall wellness (Table 4). Additionally, no influence of the level of opponents (*p* > 0.05) was evident in either of the used LMMs (Table 3 and Table 4).

## 4. Discussion

This study aimed to assess the effect of congested match schedules on match loads and well-being in addition to the effect of pre-match well-being and level of opponents on match loads in women’s rugby sevens during international tournaments. The main results showed no effect of congested match schedules on most of the investigated match load measures, while increases in fatigue and muscle soreness, which impacted overall wellness, were evident in the last day of the tournament compared to previous days. Moreover, no effect of pre-match well-being and level of opponents on match loads was found. These results overall provide useful insight for women’s rugby sevens sport scientists and practitioners, highlighting that other factors might influence players’ match loads during tournaments as well as the importance of monitoring players’ well-being status.

Our study indicated that playing several matches in close succession during the Rugby Seven’s World Series did not impact external match load intensity and internal perceived load. Indeed, no significant differences were evident in most of the internal and external match load investigated measures across matches, except for striding/min. Interestingly, these results are in line with previous research investigating external and internal load between-matches differences in women’s rugby sevens during the World Series when playing matches more frequently (four to six) in a shorter or similar time (2–3 days) [3,5]. A possible reason for these results could be the good management of post-match recovery strategies implemented by the team strength and conditioning coaches and practitioners. Another possible explanation might be that elite players possess high aerobic fitness levels, which could entail a good capacity to sustain high match loads and rapidly recover [3]. Indeed, a previous investigation of a similar-level (national) women’s rugby sevens team in comparison with a lower-level (state) team indicated that national-level players are able to sustain higher match loads with less physiological disturbance [3]. Moreover, it should be considered that players were involved with different playing times across the investigated matches due to the rotation adopted by the coaching staff. Indeed, the use of substitution might have allowed key players, who generally experience a higher match time, to recover and keep a high match intensity across the investigated matches.

Although no substantial differences were found in match loads across matches played in close succession, moderate decrements in perceived fatigue and muscle soreness values were evident across the four investigated matches, which in turn impacted overall wellness. A decrement in perceived well-being was also shown in a previous investigation assessing a national women’s rugby seven team during a tournament of the Women’s World Rugby Sevens Series [5]. However, perceived well-being was found to decrease after the first match day and remained impaired up to 2 days after the two-day tournament [5], while in our study the decrement was evident only on day four, which corresponded to the last or the post-tournament day, with no statistical changes found between previous days. A possible reason for this difference might be the dissimilar match schedules of the two investigated tournaments. Indeed, in another study [5], the impaired perceived well-being and recovery was likely due to the three matches played in the first investigated day, with two further matches played on the following day for a total of five matches in 2 days. Differently, in our investigations, players were involved in no more than two daily matches and a total of 4 matches across 2–3 days were played. This less frequent number of matches due to a different schedule of the Women’s World Rugby Sevens Series since 2018, which reduces the number of daily matches and increases recovery time, produced a beneficial effect on players’ perceived well-being, at least during the first tournament days, producing cumulative fatigue, muscle soreness and an impairment in overall wellness only toward the end of the tournaments. These results highlight that congested match schedules negatively impact perceived fatigue, muscle soreness and overall wellness. As a consequence, women’s rugby seven practitioners should monitor players’ well-being status to implement appropriate recovery strategies.

The relationships between workloads and well-being in elite women’s rugby sevens have been previously investigated, showing moderate negative correlations between high-intensity running and increased fatigue (r = −0.60; *p* = 0.049) in addition to physical contact and increased general muscle soreness (r = −0.69; *p* = 0.013) in day one of an elite women’s rugby sevens tournament [5]. While this evidence is important from a practical standpoint to identify the main match load measures affecting post-match perceived well-being [5], the assessment of the relationships between pre-match well-being and match loads could also provide further insight for women’s rugby sevens practitioners about players’ capacity to perform matches based on previous well-being values. Previous studies across various team sports mainly assessed the effect of well-being on training sessions rather than on match performance [15,16,17,18,19]. Specifically, a negative effect of pre-training perceived well-being on external load measures in Australian football players [15] and on internal perceived training load in professional male soccer [16] as well as female volleyball players [19]. Moreover, significant, although trivial, relationships were found between pre-training well-being and subsequent training load in American college football players [17]. Differently, our study was the first to assess the effect of pre-match well-being on subsequent loads, showing no effect of well-being items when analyzed singularly (Table 3) or together as overall wellness (Table 4). The differences in the results between our and previous investigations lies in the fact that many contextual factors could influence match loads compared to training loads in team sports, such as individual characteristics, team strength, opposition strength, etc. [20,21]. Therefore, monitoring pre-match well-being is important to have a clear picture of players’ status before starting the match; other contextual factors should be considered when monitoring match loads.

In an attempt to provide a multivariate analysis that includes other potential contextual factors influencing match loads, we included the level of opponents together with well-being in our analysis, which indicated no statistical effect on match loads. To date, this is the first study that assesses this contextual factor in women’s rugby sevens during tournaments, making the results hard to compare with previous investigations. However, when compared with rugby league, the overall results indicated that playing against a stronger opposition team, defined by final ladder position, produced a small increase in average speed (ES = 0.39) [22] and accelerations relative to playing time (ES = 0.21) [23]. Differently, a small increase in total distance (ES = 0.30) [23] and HSR speed (ES = 0.58–0.60) [23,24] was shown when playing with weaker teams. The difference in our results could be explained by the fact that the investigated team was classified among the low-level teams, indicating that players were competing at their maximum in any match, regardless of the opposing teams.

Although this study provides useful insights for women’s rugby sevens coaches and practitioners, some limitations should be acknowledged. Firstly, this study referred to players belonging only to one team; including a multiple-teams design might have allowed for a better generalization of the results. Additionally, a larger sample size would have also allowed a distinction between players accumulating higher or lower playing time, which might also have an influence on match loads as shown in other team sports [11,25]. Therefore, further studies are warranted on the effect of playing time on match loads and well-being in women’s rugby sevens during tournaments. Finally, although this study provided a multivariate approach investigating various contextual factors affecting match loads, further studies assessing additional contextual factors, such as players’ individual characteristics, match outcomes in addition to technical and tactical demands, which might also impact match loads in team sports [20,21].

### Practical Applications

From a practical standpoint, our results provide useful insights for women’s rugby sevens practitioners as well as strength and conditioning coaches. Firstly, during congested match schedules, it seems fundamental to monitor changes in match loads and well-being to assure that players were able to maintain their performances and well-being status during tournaments. Since changes in perceived fatigue, muscle soreness and overall wellness were documented in this study, practitioners are suggested to develop and adopt appropriate recovery strategies during and after women’s rugby sevens tournaments. Moreover, considering that no effect of pre-match well-being and level of opponents on match loads were found, it is suggested to use a multidimensional approach player monitoring system, which would include other contextual factors potentially associated with match loads.

## 5. Conclusions

This is the first study designed with a multifactorial approach to assess the effect of congested match schedules on match loads and well-being in addition to pre-match well-being and level of opponents on match loads, in elite women’s rugby sevens during tournaments. The main results indicated that a congested match schedule did not impact most of the match load investigated measures, while well-being showed a decrement in the last investigated day of the tournaments. Moreover, match loads are not influenced by pre-match well-being scores and level of opponents. These results highlighted the necessity to consider a multidimensional approach when adopting a monitoring system in elite women’s rugby sevens during tournaments, which should include both load and well-being measures as well as consider various contextual factors beside the studied ones (i.e., congested match schedule, pre-match well-being and level of opponents).

## Figures and Tables

**Table 1:** Match-to-match differences in match load measures.

Dependent Variables	AIC	R^2^ Conditional	*p*-Value
sRPE (AU)	−175.013	0.046	0.225
sRPE-ML (AU)	1097.276	0.013	0.712
TD/min (m/min)	922.071	0.277	0.483
Walking/min (m/min)	655.041	0.098	0.232
Jogging/min (m/min)	723.363	0.116	0.635
Cruising/min (m/min)	588.858	0.078	0.220
Striding/min (m/min)	700.532	0.204	0.021 #
HIR/min (m/min)	536.860	0.067	0.468
Sprint/min (m/min)	648.738	0.139	0.325
ACC/min (n/min)	120.892	0.038	0.579
DECEL/min (n/min)	170.747	0.190	0.292

Legend: LMM analysis: significant differences are presented in bold *p*-values (*p* < 0.05). Post hoc analysis: # significant difference between match 1 and match 3. AIC—Akaike information criterion; CI—confidence interval; SE—standard error; sRPE—session rating of perceived exertion; sRPE-ML—session rating of perceived exertion match load; TD/min—total distance covered per minute; walking/min—TD/min at 0–6.0 km·h^−1^; jogging/min—TD/min at 6.1–12.0 km·h^−1^; cruising/min—TD/min at 12.1–14.0 km·h^−1^; striding/min—TD/min at 14.1–18.0 km·h^−1^; HIR/min—TD/min at 18.1–20.0 km·h^−1^; sprint/min—TD/min at >20.1 km·h^−1^; ACC/min—number of accelerations per minute at >1.8 m·s^−2^; and DECEL/min—number of decelerations per minute at <−1.8 m·s^−2^.

**Table 2:** Day-to-day changes in well-being measures.

Dependent Variables	AIC	R^2^ Conditional	*p*-Value
Fatigue	−288.993	0.304	<0.001
Sleep quality	−196.895	0.139	0.043
Muscle soreness	−251.090	0.263	0.004
Stress levels	−407.567	0.780	0.275
Mood	−386.724	0.545	0.083
Overall wellness	−426.364	0.441	<0.001

Legend: AIC—Akaike information criterion.

**Table 3:** The effect of previous-day well-being and level of opponents on match load measures.

Dependent Variables	AIC	R^2^ Conditional	Fixed Effects	Estimate (95% CI)	SE	*p*-Value
sRPE (AU)	−113.336	0.054	Fatigue	0.017 (−0.322, 0.355)	0.173	0.924
			Sleep	−0.136 (−0.405, 0.134)	0.138	0.328
			Soreness	−0.085 (−0.039, 0.217)	0.154	0.584
			Stress	−0.079 (−0.470, 0.312)	0.199	0.694
			Mood	−0.262 (−0.728, 0.203)	0.237	0.273
			Level of opponents	0.008 (−0.039, 0.056)	0.024	0.736
sRPE-ML (AU)	754.650	0.100	Fatigue	74.970 (−65.500, 215.480)	71.690	0.299
			Sleep	−113.260 (−225.100, −1.430)	57.060	0.051
			Soreness	−54.810 (−180.100, 70.510)	63.940	0.394
			Stress	−112.100 (−274.100, 49.900)	82.650	0.179
			Mood	−37.190 (−230.200, 155.780)	98.450	0.707
			Level of opponents	3.620 (−16.100, 23.310)	10.040	0.719
TD/min (m/min)	634.595	0.232	Fatigue	−13.939 (−73.370, 45.490)	30.320	0.647
			Sleep	−10.443 (−57.040, 36.150)	23.770	0.662
			Soreness	−22.209 (−74.960, 30.540)	26.910	0.412
			Stress	24.807 (−42.800, 92.420)	34.490	0.475
			Mood	15.841 (−63.020, 94.710)	40.240	0.695
			Level of opponents	0.584 (−7.440, 8.610)	4.090	0.887
Walking/min (m/min)	446.211	0.046	Fatigue	−5.772 (−22.270, 10.730)	8.418	0.495
			Sleep	4.047 (−9.080, 17.180)	6.700	0.548
			Soreness	−6.603 (−21.320, 8.110)	7.508	0.382
			Stress	0.258 (−18.770, 19.280)	9.706	0.979
			Mood	0.180 (−22.480, 22.840)	11.561	0.988
			Level of opponents	1.224 (−1.090, 3.540)	1.179	0.303
Jogging/min (m/min)	497.060	0.132	Fatigue	−8.290 (−31.550, 14.980)	11.870	0.487
			Sleep	−4.650 (−23.010, 13.700)	9.360	0.621
			Soreness	−3.910 (−24.560, 16.740)	10.540	0.712
			Stress	13.680 (−12.950, 40.320)	13.590	0.318
			Mood	−4.880 (−36.180, 26.430)	15.970	0.761
			Level of opponents	1.020 (−2.170, 4.200)	1.630	0.534
Cruising/min (m/min)	415.451	0.026	Fatigue	−2.035 (−15.360, 11.290)	6.799	0.766
			Sleep	−3.774 (−14.380, 6.830)	5.411	0.488
			Soreness	−1.830 (−13.720, 10.060)	6.064	0.764
			Stress	4.776 (−10.590, 20.140)	7.839	0.544
			Mood	1.457 (−16.840, 19.760)	9.338	0.876
			Level of opponents	0.157 (−1.710, 2.020)	0.952	0.869
Striding/min (m/min)	481.644	0.206	Fatigue	5.930 (−14.740, 26.605)	10.550	0.576
			Sleep	−3.020 (−19.250, 13.217)	8.280	0.717
			Soreness	−3.270 (−21.620, 15.068)	9.360	0.728
			Stress	13.390 (−10.170, 36.955)	12.020	0.269
			Mood	3.170 (−24.370, 30.703)	14.050	0.823
			Level of opponents	−1.880 (−4.690, 0.917)	1.430	0.192
HIR/min (m/min)	379.950	0.086	Fatigue	−6.593 (−17.010, 3.820)	5.313	0.219
			Sleep	−1.592 (−9.880, 6.700)	4.229	0.708
			Soreness	−3.325 (−12.610, 5.960)	4.739	0.485
			Stress	5.204 (−6.800, 17.210)	6.126	0.398
			Mood	5.654 (−8.650, 19.960)	7.297	0.441
			Level of opponents	0.115 (−1.340, 1.570)	0.744	0.877
Sprint/min (m/min)	445.249	0.146	Fatigue	−0.144 (−16.330, 16.040)	8.258	0.986
			Sleep	−1.463 (−14.210, 11.280)	6.504	0.823
			Soreness	−7.444 (−21.800, 6.920)	7.327	0.313
			Stress	−11.816 (−30.320, 6.680)	9.439	0.215
			Mood	14.583 (−7.120, 36.280)	11.071	0.192
			Level of opponents	0.523 (−1.680, 2.730)	1.126	0.644
ACC/min (n/min)	99.437	0.098	Fatigue	0.387 (−1.092, 1.866)	0.755	0.610
			Sleep	0.414 (−0.757, 1.586)	0.598	0.491
			Soreness	−0.805 (−2.120, 0.510)	0.671	0.234
			Stress	−0.377 (−2.076, 1.323)	0.867	0.665
			Mood	1.422 (−0.586, 3.430)	1.025	0.170
			Level of opponents	−0.010 (−0.214, 0.195)	0.104	0.926
DECEL/min (n/min)	136.354	0.148	Fatigue	−0.617 (−2.535, 1.302)	0.979	0.531
			Sleep	−0.258 (−1.269, 1.785)	0.779	0.741
			Soreness	−1.657 (−3.368, 0.055)	0.873	0.062
			Stress	1.428 (−0.784, 3.640)	1.129	0.210
			Mood	0.959 (−1.676, 3.593)	1.344	0.478
			Level of opponents	0.031 (−0.238, 0.300)	0.137	0.822

Legend: AIC—Akaike information criterion; CI—confidence interval; SE—standard error; sRPE—session rating of perceived exertion; sRPE-ML—session rating of perceived exertion match load; TD/min—total distance covered per minute; walking/min—TD/min at 0–6.0 km·h^−1^; jogging/min—TD/min at 6.1–12.0 km·h^−1^; cruising/min—TD/min at 12.1–14.0 km·h^−1^; striding/min—TD/min at 14.1–18.0 km·h^−1^; HIR/min—TD/min at 18.1–20.0 km·h^−1^; sprint/min—TD/min at > 20.1 km·h^−1^; ACC/min—number of accelerations per minute at >1.8 m·s^−2^; and DECEL/min—number of decelerations per minute at <−1.8 m·s^−2^.

**Table 4:** Effect of previous-day overall wellness and level of opponents on match load measures.

Dependent Variables	AIC	R^2^ Conditional	Fixed Effects	Estimate (95% CI)	SE	*p*-Value
sRPE (AU)	−120.659	0.045	Overall wellness	−0.535 (−1.111, 0.041)	0.294	0.073
			Level of opponents	0.007 (−0.040, 0.054)	0.024	0.776
sRPE-ML (AU)	750.707	0.047	Overall wellness	−229.910 (−474.300, 14.500)	124.68	0.069
			Level of opponents	3.600 (−16.400, 23.600)	10.190	0.725
TD/min (m/min)	629.666	0.237	Overall wellness	−15.177 (−122.150, 91.800)	54.580	0.782
			Level of opponents	−0.268 (−8.260, 7.730)	4.080	0.948
Walking/min (m/min)	440.221	0.018	Overall wellness	−6.610 (−34.900. 21.690)	14.435	0.649
			Level of opponents	1.270 (−1.040, 3.580)	1.179	0.284
Jogging/min (m/min)	491.835	0.120	Overall wellness	−16.350 (−57.670, 24.970)	21.080	0.441
			Level of opponents	0.635 (−2.540, 3.810)	1.620	0.697
Cruising/min (m/min)	408.974	0.020	Overall wellness	−5.602 (−28.520, 17.320)	11.696	0.634
			Level of opponents	−0.006 (−1.850, 1.840)	0.942	0.995
Striding/min (m/min)	475.585	0.221	Overall wellness	11.860 (−24.960, 48.675)	18.790	0.530
			Level of opponents	−2.220 (−4.990, 0.547)	1.410	0.121
HIR/min (m/min)	377.979	0.024	Overall wellness	−4.588 (−23.090, 13.920)	9.442	0.629
			Level of opponents	−0.015 (−1.500, 1.470)	0.759	0.984
Sprint/min (m/min)	440.206	0.111	Overall wellness	−0.774 (−29.640, 28.090)	14.728	0.958
			Level of opponents	0.696 (−1.530, 2.920)	1.134	0.541
ACC/min (n/min)	94.742	0.061	Overall wellness	1.432 (−1.173, 4.037)	1.329	0.285
			Level of opponents	0.003 (−0.203, 0.209)	0.105	0.975
DECEL/min (n/min)	137.087	0.136	Overall wellness	0.297 (−3.221, 3.815)	1.795	0.869
			Level of opponents	−0.011 (−0.279, 0.258)	0.137	0.937

Legend: AIC—Akaike information criterion; CI—confidence interval; SE—standard error; sRPE—session rating of perceived exertion; sRPE-ML—session rating of perceived exertion match load; TD/min—total distance covered per minute; walking/min—TD/min at 0–6.0 km·h^−1^; jogging/min—TD/min at 6.1–12.0 km·h^−1^; cruising/min—TD/min at 12.1–14.0 km·h^−1^; striding/min—TD/min at 14.1–18.0 km·h^−1^; HIR/min—TD/min at 18.1–20.0 km·h^−1^; sprint/min—TD/min at >20.1 km·h^−1^; ACC/min—number of accelerations per minute at >1.8 m·s^−2^; DECEL/min—number of decelerations per minute at <−1.8 m·s^−2^.

## Data Availability

The data presented in this study are available on request from the corresponding author. The data are not publicly available due to privacy reasons.
